# MRI in Late-Onset Rasmussen Encephalitis: A Long-Term Follow-Up Study

**DOI:** 10.3390/diagnostics12020502

**Published:** 2022-02-15

**Authors:** Fabio Martino Doniselli, Francesco Deleo, Stefania Criscuolo, Andrea Stabile, Chiara Pastori, Roberta Di Giacomo, Giuseppe Didato, Luisa Chiapparini, Flavio Villani

**Affiliations:** 1 Neuroradiology Unit, Fondazione IRCCS Istituto Neurologico Carlo Besta, Via Celoria 11, 20133 Milan, Italy; fabio.doniselli@istituto-besta.it (F.M.D.); luisa.chiapparini@istituto-besta.it (L.C.); 2 Department of Biomedical Sciences for Health, University of Milan, Via Mangiagalli 31, 20122 Milan, Italy; 3 Epilepsy Unit, Fondazione IRCCS Istituto Neurologico Carlo Besta, Via Celoria 11, 20133 Milan, Italy; andrea.stabile@istituto-besta.it (A.S.); chiara.pastori@istituto-besta.it (C.P.); roberta.digiacomo@istituto-besta.it (R.D.G.); giuseppe.didato@istituto-besta.it (G.D.); 4 Postgraduational School of Radiodiagnostics, University of Milan, Via Festa del Perdono 7, 20122 Milan, Italy; stefania.criscuolo@unimi.it; 5 Division of Clinical Neurophysiology and Epilepsy Center, IRCCS Ospedale Policlinico San Martino, Largo R. Benzi 10, 16132 Genoa, Italy; flavio.villani@hsanmartino.it

**Keywords:** Rasmussen encephalitis, MRI, late-onset, epilepsy

## Abstract

Late-onset Rasmussen encephalitis (LoRE) is a rare unihemispheric progressive inflammatory disorder causing neurological deficits and epilepsy. The long-term radiological evolution has never been fully described. We retrospectively analyzed the MR images of 13 LoRE patients from a total of 136 studies, and searched for focal areas of volume loss or signal intensity abnormality in grey matter or white matter. Each subject had a median of nine MRI studies (IQR 7–13). Frontal and temporal lobes were the most affected regions (13/13 and 8/13, respectively) and showed the greatest worsening over time in terms of atrophic changes (9/13 and 5/8, respectively). A milder cortical atrophy was found in the insular and parietal lobes. The caudate nucleus was affected in seven patients. Hyperintensities of grey matter and white matter on T2-WI and FLAIR images were observed in all patients, and transiently in eight patients. In two cases out of the latter patients, these transient alterations evolved into atrophy of the same region. Disease duration was significantly associated with signal abnormalities in the grey matter at last follow-up. LoRE MRI alterations are milder, and their progression is markedly slower compared to radiological findings described in the childhood form.

## 1. Introduction

Rasmussen encephalitis (RE) is a rare unihemispheric progressive inflammatory disorder. The estimated incidence, determined in Germany, is about two cases per 10 million people aged 18 years and younger, per year [1]. RE usually affects children, but a late-onset RE (LoRE) variant, accounting for about 10% of all cases, has been described. LoRE patients present a milder and slower evolution, involving less-severe neurological deficits [2,3,4].

RE etiology is still unknown. In their original paper [5], Rasmussen and coauthor suggested a “viral” etiology. However, clear signs of viral infection were neither confirmed nor definitely excluded [6]. The characteristic findings in RE histopathology are inflammation, neuronal loss and gliosis. Inflammation is multifocal within one hemisphere and progressive. In more severe cases, cortical cavitation, marked astrogliosis, and neuronal cell loss are also present. This pattern suggests an immune-mediated disease [5,6,7]. T-cell-mediated cytotoxicity has been implicated as one of the main mechanisms of RE pathogenesis [8,9,10]. 

RE diagnostic criteria were established by a panel of experts in 2005 [11]. The syndrome is characterized by focal seizures; unihemispheric slowing and unilateral seizure onset on electroencephalogram (EEG) and progressive unihemispheric focal atrophy with signal alteration on magnetic resonance imaging (MRI). Epilepsia partialis continua (EPC), first described by the Russian neurologist Kozhevnikov in the late 19th century [12], is a hallmark of the disease. In some cases, a brain biopsy is necessary to confirm the diagnosis. 

The aim of RE treatment is twofold: controlling both seizures and disease atrophic progression. RE treatment relies on antiseizure medications (ASMs), immune therapy and surgery. Seizures are often drug-resistant, and EPC is usually refractory to all ASMs. Different immune therapies have been proposed [13,14,15]. The main aim of immune therapy is stopping disease progression. The treatment of choice for RE is functional hemispherectomy, and it is highly effective on epilepsy and its consequences [14,16,17]. This surgical treatment option, however, may not be feasible in patients with preserved neurologic function because of the severe deficits it may induce, particularly when surgery is performed in adolescent or adult patients, when the brain plasticity and the possible recovery are limited. A limited cortical resection, in selected cases, may be taken into account in those cases [18].

Brain MRI shows unilateral progressive cortical atrophy, atrophy of the head of the caudate nucleus and abnormal signal intensity, mainly in the subcortical white matter beneath the same region of cortical atrophy, leading to an hemispheric volume loss [19]. Most of these MRI features studies, however, concern the pediatric population, whereas evidence about imaging findings in adults is substantially lacking.

The aim of this study is to describe LoRE MRI presentation and to semiquantitatively evaluate disease progression. 

## 2. Materials and Methods

### 2.1. Patient Cohort

The study includes 13 patients with a LoRE diagnosis according to the European Consensus Criteria [11] collected from 2006 to 2021. Clinical data (medical history, neurologic examination, neuropsychological testing), EEG, video-EEG, MRI and treatment options were available in all patients. In four patients, a histological analysis confirmed the diagnosis.

All diagnostic and therapeutic procedures were decided according to the standard clinical care of our Institute. 

All patients were followed at the Epilepsy Unit of the Foundation IRCCS Neurological Institute “C. Besta” of Milan. 

### 2.2. Radiological Analysis 

Patients’ images are stored in the Institute’s digital database (PACS). Inclusion criteria were the presence of T1-weighted and T2-weighted or FLAIR images to assess the degree of atrophy and the presence of hyperintensity of intracranial structures, respectively.

MRI scans were performed using different scanners: a 1.5 T Siemens Avanto Fit, 1.5 T Philips Achieva or 3 T Philips Achieva Dstream. During this 17-year follow-up, sequences and protocols changed over time (from T1-SE images 4–5 mm of thickness of the first studies to the more recent volumetric T1-FFE 0.76 mm of thickness). Images were of sufficient quality for evaluation in all cases.

Two neuroradiologists (a radiology resident with 2 years of experience in neuroradiology, S.C., and a neuroradiologist with 6 years of experience, F.M.D.) analyzed the images autonomously. All discordances were discussed, and a final consensus reached. The brain areas were divided into the two hemispheres and different sublobar regions, as proposed by previous study [20], in particular: the frontal lobe (anterior frontal, inferior frontal, mesial frontal, frontal operculum, precentral), cingulum, parietal lobe, thalamus, striatum, caudate nucleus, temporoinsular lobe (insula, perisylvian temporal, anterior temporal, mesial temporal, hippocampus) and occipital lobe (trigone, occipital, mesial occipital). 

Grade of cortical atrophy and signal abnormalities of grey matter (GM) and white matter (WM) were scored in a semiquantitative manner between 0 and 3 (0, no involvement; 1, mild involvement; 2, moderate involvement; 3, severe involvement). 

## 3. Results

We analysed 13 patients diagnosed with LoRE. Median age of symptom onset was 25 years (IQR 19–32 years) and median disease duration was 18 years (IQR 7–21 years). Symptoms at onset were seizures (9 out of 13 patients), neurological focal deficits (7/13), EPC (5/13), cognitive deficits (1/13) and behavioural (1/13) alterations. Clinical features at last follow-up were seizures (10/13), neurological focal deficits (13/13), EPC (7/13), cognitive deficits (8/13) and behavioural (8/13) alterations.

A dataset of 136 MRI images was included in this study, with a median of nine MRI controls each (IQR 7–13). Overall median time between follow-ups was 7 months (IQR 4.4–13 months), with differences between patient (see Table 1 and Table S1).

Early MRI (within 2 years from symptoms onset) was performed in six patients (46%); in two patients the time between symptom onset and first available MRI was between 20 and 30 years (patients 7 and patient 13); all the other 5 patients had a first MRI between 3 and 12 years from symptom onset. 

Among the patients with an early MRI performed (within 2 years from onset), volume loss was prevalent in the frontal lobe, with mild (four out of six patients) or moderate (two out of six patients) alterations. An alteration in the temporal and parietal lobes was also visible from the first scan (in four and two out of six patients, respectively). The signal alterations were present at the onset in five of the six patients, both of the WM and of the GM, in the same regions affected by atrophic involution.

A histological analysis is available in four cases (patients 3, 6, 8 and 12). In one case (patient 12), surgery was performed to confirm the diagnosis and not to treat epilepsy. The remaining three cases (patients 3, 6 and 8) underwent a cortical resection limited to the epileptogenic zone (for both seizures and EPC). Outcome was good in patients 3 and 8: they had a significant improvement in both seizures and mainly in EPC [18], but outcome was poorer in the last operated case (patient 6), likely because of incomplete resection of the epileptogenic zone. 

In the contralateral, not-affected cerebral hemisphere, only subtle volume loss was seen in two cases (patient 10 and 13); no signal abnormality was ever found. In the first examinations, when gadolinium was administered, no meningeal or cerebral contrast enhancement was observed.

### 3.1. Atrophic Changes

**Figure 1:**
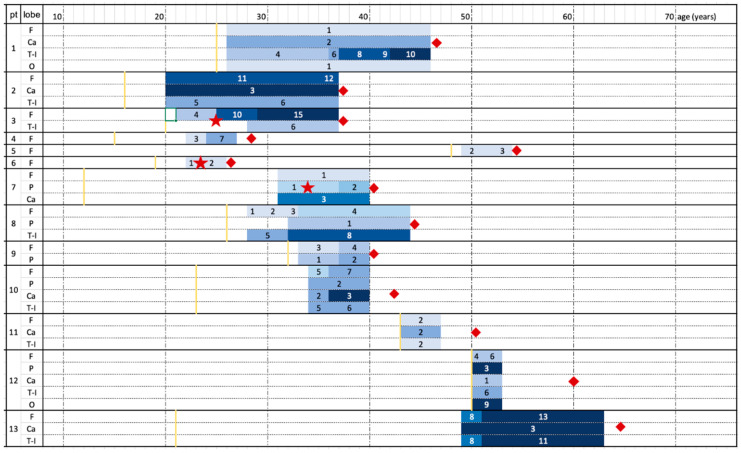
Atrophy progression timeline for each patient. Patients’ age (years) increases from left to right. The shades of blue represent MRI controls and worsening of the degree of atrophy (according to numerical values deriving from the sum of the scores of different areas, as mentioned in Table 2). Yellow line represents clinical onset; red squares represent patients’ last clinical evaluation and red stars the surgical intervention. Patient 10 and 13 died after last follow-up due to non-neurological disease. F = frontal lobe; Ca = Caudate nucleus; T-I = temporoinsular lobes; P = parietal lobe; O = occipital lobe.

**Figure 2:**
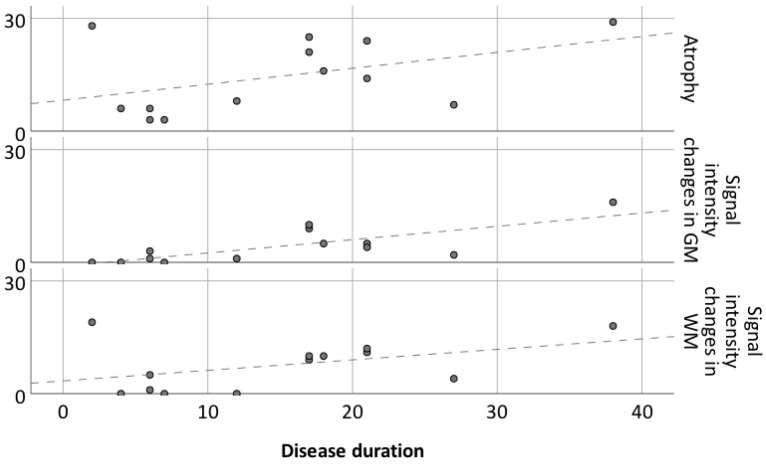
Atrophy and signal intensity changes in gray and white matter in relation to disease duration for 13 patients. Dashed lines represent linear regression for atrophy, GM SI changes and WM SI changes.

**Figure 3:**
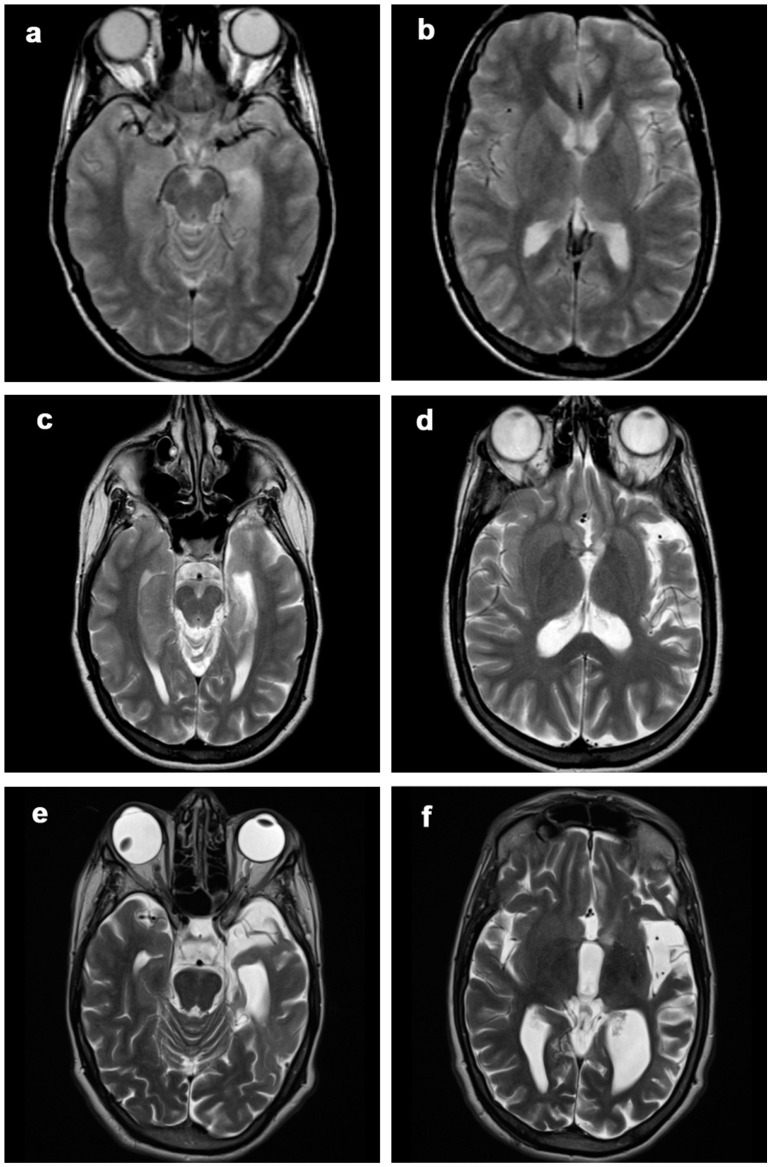
Patient 1, progression of the disease in 21 years. Axial (**a**–**f**) T2-weighted images at (**a**,**b**) 2 years, (**c**,**d**) 12 years and (**d**,**e**) 21 years from clinical onset. The disease slowly progressed over time, involving mainly frontobasal, insular, perisylvian and temporal left structures. At first MRI scan, only subtle atrophic changes could be seen in the perisylvian region (**b**).

Frontal and temporoinsular lobes were the most affected (13/13 and 8/13, respectively, Figure 1, Table 2) and showed the greatest worsening over time in terms of atrophic changes (9/13 and 5/8, respectively, Figure 2). Even if the frontal lobe is globally more affected, the cortical atrophy of the temporal and insular lobe is more marked. The caudate nucleus was affected in seven patients (in patient 12 and patient 1—Figure 3—and patient 11 with mild and moderate atrophic changes; in four patients with severe involvement). The parietal lobe was affected in five patients (two showing atrophic progression). The occipital lobe was only affected in one patient.

At the first MRI check, all patients had at least one area affected by volume loss.

**Figure 4:**
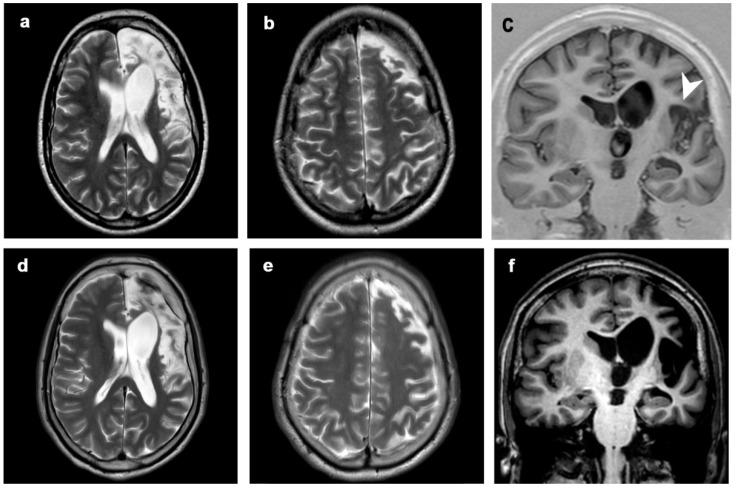
Patient 2, progression of the disease in 21 years. Axial (**a**,**b**,**d**,**e**) T2-weighted images, coronal T1-inversion recovery (**c**) and T1-weighted image (**f**) at (**a**–**c**) 5 years (first MRI scan) and (**d**–**f**) 21 years from clinical onset (last MRI scan). The disease showed mild progression over time, involving left perisylvian region (including frontal, insular and temporal structures, arrowhead). Clinical symptoms progressed from seizures at onset to seizures, focal deficits, EPC, cognitive and behavioral disorders.

**Figure 5:**
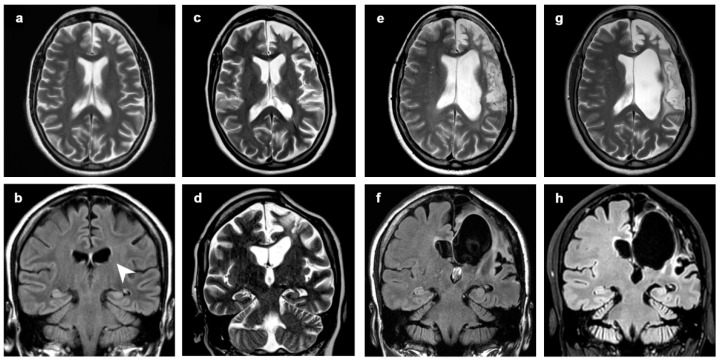
Patient 3, progression of the disease in 17 years. Axial (**a**,**c**,**e**,**g**) and coronal (**d**) T2-weighted images, coronal FLAIR (**b**,**f**,**h**) at (**a**,**b**) 2 years, (**c**,**d**) 5 years, (**e**,**f**) 10 years and (**g**,**h**) 17 years from clinical onset. In the first MRI (**a**,**b**), only subtle dilatation of left ventricle (arrowhead) and enlargement of frontal sulci could be seen. In (**d**), WM hyperintensity preceded the later atrophic change. Surgical treatment was performed at 6 years from onset, between (**c**,**d**) and (**e**,**f**); after surgery, EPC was controlled. At last follow-up, seizures and cognitive and behavioral disorders were present.

**Figure 6:**
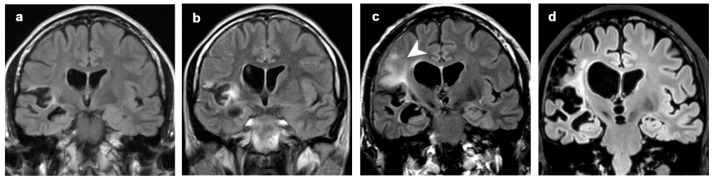
Patient 8, hyperintense signal intensities that preceded focal atrophy. Coronal FLAIR images at (**a**) 3 years, (**b**) 4 years, (**c**) 8 years and (**d**) 18 years from clinical onset. Focal atrophy limited to right perisylvian and insular region at the first MRI scan was followed by hyperintense alteration both of GM and WM in insula and frontal lobe (**b**,**c**, arrowhead). These alterations preceded worsening of the atrophic changes in the same areas.

**Figure 7:**
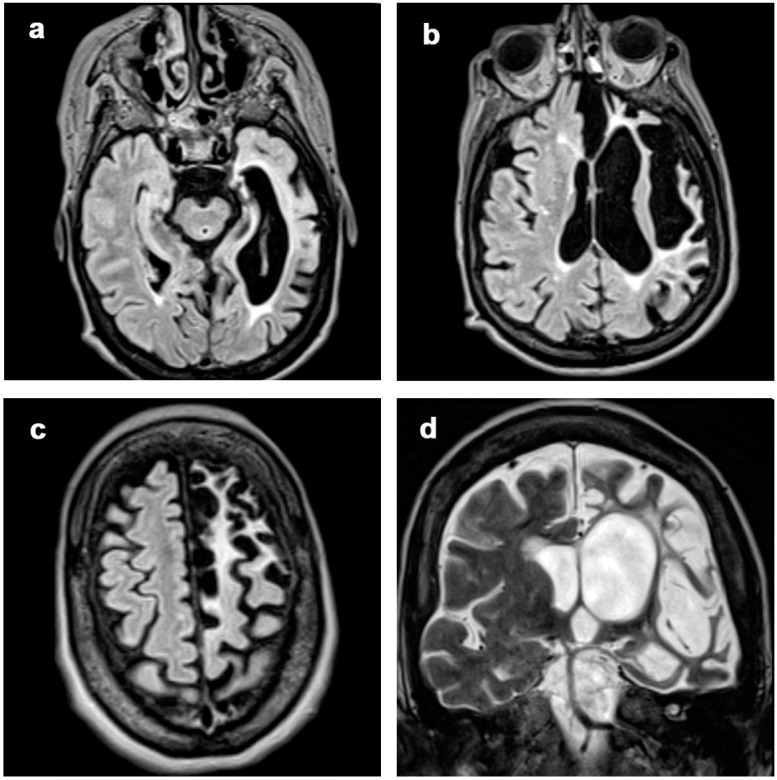
Patient 13, with stable atrophy changes during 12 years of MRI controls. Axial FLAIR images (**a**–**c**) and coronal T2-weighted image (**d**) at a single timepoint, showing frontal, insular and temporal atrophy with SI involving subcortical WM and cortical GM. The patient has a 25 years of disease duration with clinical onset with focal deficits, seizures and cognitive disorders; at last follow-up, EPC and behavioral disorders are present.

Atrophy progression was clearly present in most patients (Figure 3, Figure 4, Figure 5, Figure 6 and Figure 7). However, it was absent for 1 patient out of 13 (patient 11, MRI follow-up for at least 5 years) and only minimal progression was seen for patient 5 (6 years of radiological follow-up), for patient 6 (4 years of radiological follow-up) and for patient 12 (3 years of radiological follow-up). The first MRI of patient 13 (Figure 7) available in our digital repository dates 24 years after 

The onset of the disease, when the disease progression was already at the residual stage [11]; from this examination to the last MRI check (14 years of follow-up), only a minimal progression of volume loss is visible in the areas already affected. No clear relationship was found between the progression rate and age at onset, severity at onset, time to immune therapy or clinical phenotype. 

**Figure 8:**
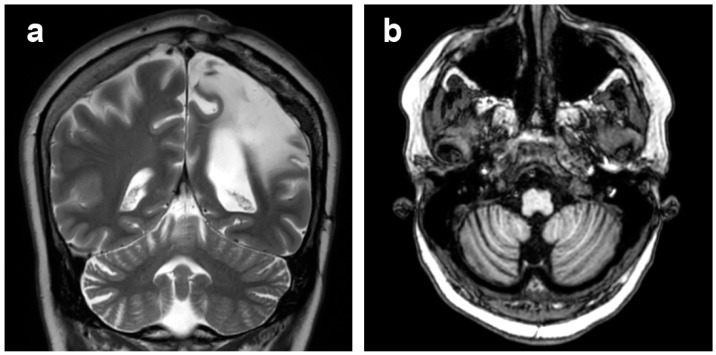
Patient 1, cerebellar diaschisis. (**a**) Coronal T2-weighted image and (**b**) axial T1-weighted image, showing right cerebellar atrophy contralateral to the affected cerebral hemisphere.

There is a tendency of association between disease duration and atrophy severity, as shown in Figure 1 and Figure 2. It is worth noting that atrophy also progressed after surgery in patients 3 (Figure 5) and 8 (Figure 6), despite their positive seizure and EPC outcome.

Moderate or severe atrophic changes in the cerebellar hemispheres contralateral to the affected cerebral hemisphere (cerebellar diaschisis, Figure 8) were observed at the last MRI scan in 11 patients. No cerebellar signal abnormality was seen. 

### 3.2. Changes in Signal Intensity

Hyperintensities of GM and WM on T2-WI and FLAIR images were seen in all patients (Table 3, Figure 3, Figure 4, Figure 5, Figure 6 and Figure 7). The degree of atrophy was the predominant finding compared to the signal intensity alterations in seven patients. In five patients, a marked degree of atrophy (score 3) was accompanied by a high degree of T2 / FLAIR hyperintensity (score 3) in the last MRI follow-up. In only one patient (patient 5) were the degree of atrophy mild and the signal changes moderate (score 1 and 2, respectively).

At the first MRI scan, two patients had no WM or GM signal changes. All the others had at least one subtle alteration at onset. At the last MRI follow-up, these changes affected the WM more than GM in five patients; the same degree of involvement (GM as WM) was described in seven patients. Only one patient at the last follow-up showed mild hyperintensities in GM and no alterations in WM (patient 4).

The presence of transitory hyperintense changes was demonstrated in eight patients: in three patients these changes involved both WM and GM; in four patients only GM; in one patient only the WM. In two cases (patient 3 and patient 8) these transient alterations (of severe degree and undefined duration in patient 3, Figure 5; of moderate degree and long duration in patient 8, Figure 6) evolved into atrophy of the same region. In the remaining six patients, these changes were graded 1 to 3 but of short duration (one MRI control only) and did not lead to atrophy.

We found an association between signal abnormalities and disease duration (Figure 2). In particular, we found a significant association between disease duration and severity of signal intensity changes in the grey matter (R = 0.58, *p* = 0.002, Figure 2).

## 4. Discussion

We analyzed MRIs of 13 patients affected by LoRE, with radiological follow-ups performed over a time span ranging from 3 to 19 years. In all our patients, MRI showed focal or diffuse signal changes, with variable degree of progressive hemiatrophy, involving both superficial and deep structures, with enlargement of the lateral ventricle and sulci, confined to one hemisphere. Both signal alteration and hemiatrophy progressed over time in almost all cases. The rate of disease progression on MRI is heterogeneous and not clearly related to the clinical phenotype or therapy. It is worth noting that even when seizures were almost controlled, after surgical cortical resection, atrophy and signal alteration still progressed over time (patients 3 and 8). The heterogeneity of both the clinical and radiological picture in a such rare disease makes it very difficult to recognize risk factors of disease progression, and further studies with more numerous recruited patients are needed.

As observed in pediatric cases, in our cohort the main finding is the presence of a progressive unilateral hemispheric atrophy involving one or more brain areas. The evidence of minimal focal atrophy located in specific areas is considered a rare radiological finding, and its presence may represent an important hint for suspecting RE in adult patients. The main differential diagnosis, including other autoimmune disorders or viral encephalitis, and vasculitis, may be ruled out due to different clinical presentations, cerebrospinal fluid analysis findings, disease course [21,22], or gadolinium enhancement.

It is already reported that LoRE is a milder and slower variant of RE compared to the childhood form, from both clinical and neuroradiological points of view [3]. The progression of atrophic changes and the appearance of hyperintense signal alterations in our adult cases, despite the variability between the different subjects, showed a slower course than that observed in pediatric series [19,23]. While in the latter the disease showed a violent evolution within the first few years from the onset, our patients had a progression from mild to severe even over the course of 20 years.

In LoRE cases, the involvement of the head of the caudate nucleus is less prominent than in the pediatric cases [19,23,24], occurring in only 6 of the 13 patients analyzed. Similarly to what is observed in pediatric patients, the frontal, temporal and insular areas are the most prominently involved. To be noted, we also found a relatively high involvement of the parietal and occipital lobes (six patients), as already reported by different authors [2,8]. However, only in patient 12 was the posterior involvement relevant from disease onset, and in the remaining cases it appeared during the advanced disease course.

Hyperintense alterations in T2 / FLAIR images are present ubiquitously in all patients with different grades of severity. Transitory hyperintense changes were found in eight patients, but only in two patients they preceded the onset of atrophy in the same region. The discriminating process underlying the two events (transitory hyperintense alterations and appearance of atrophy) can be linked to two different mechanisms: metabolic and inflammatory. The former alludes to hyperintense changes especially in GM (following the cortical ribbon) and less frequently in WM, due to sustained ictal activity [25,26]. In our study this mechanism could explain the transitory changes in one single MRI scan. However, the majority of hyperintensities are multiple and located in the subcortical area without precisely following the cortical ribbon. They are likely related to the inflammatory pathogenetic process, heralding a later possible development of local atrophy.

We found mild atrophy in the contralateral hemisphere and cerebellar alterations in our cohort, always without notable signal alterations. A previous semiquantitative radiological study [27] confirms subtle volume loss in the contralateral unaffected hemisphere. In particular, the minimal contralateral volume loss was found only in two patients with higher disease duration and more severe homolateral volume loss. According to previous studies [19,28], we endorse the hypothesis of axonal loss through the corpus callosum and through projections to posterior fossa structures, rather than a bilateral asymmetric primary disease.

Despite notable advances in the technique, the morphological criterion and the conventional MR examination—meaning atrophy and hyperintensities involving WM and GM—remain the cornerstones of radiological evaluation, as it was 20 years ago, both in children and adults [29].

Our study has some limitations. First, intrapatient MRI protocols across the years are not homogeneous, and therefore the radiological analyses were performed in a semiquantitative manner by two neuroradiologists in consensus, not allowing for automated methods (mostly due to lacking volumetric T1-weighted images). Second, radiological follow-up periods are not uniform, varying consistently from a minimum of 2 to a maximum of 21 years. Third, patients’ therapeutic histories are heterogeneous in relation to both medical and surgical procedures.

## 5. Conclusions

In LoRE patients, unihemispheric temporoinsular and frontal atrophy constitutes a major finding, with less involvement of other brain areas and inconstant atrophy of the head of the caudate nucleus. T2-WI and FLAIR image hyperintensities of GM and WM are always prominent in the affected hemisphere, in some cases in a transient fashion, and they progress over time. T2-WI and FLAIR signal hyperintensities are generally less pronounced than brain atrophy. Both atrophic and hyperintense alterations have a long-lasting course, with a progression from mild to severe, even over extremely long periods such as decades, regardless of surgical or medical therapy. Brain MRI is critical in order to follow the progression of these aspects over time.

## Figures and Tables

**Table 1:** Clinical characteristics of the population.

Patient (Sex)	Age at Onset (Symptom)	Disease Duration (Years)	Clinical Features at Last Follow-Up	Immunotherapy	Age at Surgery
1 (F)	25 (seizures, behavioural disorder)	20	Focal deficit, seizures, cognitive and behavioural disorder	Steroids, IvIg, AZA, MMF	-
2 (F)	16 (seizures)	21	Focal deficit, seizures, EPC, cognitive and behavioural disorder	Steroids, IvIg, PAI, MTX, MMF	-
3 (F)	20 (focal deficit, EPC, seizures)	17	Focal deficit, seizures, cognitive and behavioural disorder	Steroids, IvIg, PAI, MTX, PEX, AZA, RTX	25
4 (M)	15 (EPC)	28	Focal deficit, seizures, EPC, cognitive and behavioural disorder	Steroids, IvIg, MMF	-
5 (M)	48 (focal deficit, EPC, seizures)	5	Focal deficit, EPC, seizures	Steroids, IvIg, MMF	-
6 (F)	19 (EPC, seizures)	7	Focal deficit, EPC, seizures	Steroids, IvIg, AZA	24
7 (F)	12 (focal deficit, seizures)	27	Focal deficit, seizures, cognitive and behavioural disorder	Steroids, IvIg, MMF	-
8 (F)	26 (focal deficit, seizures)	18	Focal deficit	Steroids, IvIg, AZA, PEX,	33
9 (M)	33 (focal deficit, EPC)	7	Focal deficit, EPC, seizures	Steroids, IvIg, AZA	-
10 (F)	23 (seizures)	18 *	Focal deficit, seizures, EPC, cognitive and behavioural disorder	Steroids, IvIg, MTX, AZA, cyclophosphamide	-
11 (M)	43 (status epilepticus)	5	Focal deficit	Steroids, IvIg	-
12 (F)	50 (focal deficit)	5	Focal deficit, cognitive and behavioural disorder	Steroids, IvIg	50
13 (F)	21 (focal deficit, seizures, cognitive disorder)	25 *	Focal deficit, seizures, EPC, cognitive and behavioural disorder	Steroids, IvIg, PEX, PAI	-

EPC: epilepsia partialis continua; IvIg: intravenous immunoglobulin; AZA: azathioprine; MMF: mycophenolate mofetil, PAI: protein A immune-absorption, MTX: mitoxantrone, PEX: plasma exchange, RTX: rituximab. * deceased for causes other than RE.

**Table 2:** Brain atrophy of 13 patients with Rasmussen encephalitis. Each row represents radiological findings when any change occurred (either in atrophy or in signal intensity); first and last MR exam details are always reported.

Patient (Affected Side)	No. Of MRI Examinations	Time between Follow-Ups (Months, Mean ± DS)	MRI Timing		MRI Findings
			Patient Age	Years from Onset	Atrophy on T1
1 (L)	13	19 ± 30	27	2	Fi (1), Ca (2), I (2),ps (1),Tm (1)
			37	12	Fi (1), Ca (2), I (3), ps (1), Tp (1), Tm (1),Ti (1)
			38	13	Fi (1), Ca (2), I (3), ps (2), Tp (1), Tm (2), Ti (1)
			42	17	Fi (1), Ca (2), I (3), ps (2), Tp (2), Tm (2), Ti (1)
			43	18	Fi (1), Ca (2), I (3), ps (3), Tp (2), Tm (2), Ti (1)
2 (L)	14	15 ± 14	21	5	Fi (3), Fm (3), F (3), Op (2), Ca (3), pv (2), I (2), Tp (1), ps (2)
			25	9	Fi (3), Fm (3), F (3), Op (2), Ca (3), pv (2), I (3), Tp (1), ps (2)
			30	14	Fi (3), Fm (3), F (3), Op (2), Ca (3), pv (3), I (3), Tp (1), ps (2)
			37	21	Fi (3), Fm (3), F (3), Op (2), pc (1), Ca (3), pv (3), I (3), Tp (1), ps (2)
3 (L)	17	11 ± 12	22	2	F (1), Fi (1), Fm (1), Op (1), pv (1)
			24	4	F (1), Fi (1), Fm (1), Op (1), pv (1)
			25	5	F (2), Fi (2), Fm (2), Op (2), pv (2)
			29	9	F (3), Fi (3), Fm (3), Op (3), pv (3), I (2), Tm (1), Tp (1), ps (1)
			30	10	F (3), Fi (3), Fm (3), Op (3), pv (3), I (2), Tm (1), Tp (1), ps (2)
			34	14	F (3), Fi (3), Fm (3), Op (3), pv (3), I (3), Tm (1), Tp (1), ps (2)
4 (R)	8	7 ± 5	23	8	F (1), Fm (1), Op (1), pv (1)
			25	10	F (2), Fm (2), Op (2), pv (2), pc (1)
5 (R)	9	6 ± 3	50	2	F (1), pc (1)
			53	5	F (1), pc (2)
6 (L)	10	5 ± 4	23	4	pc (1)
			24	5	pc (2)
			26	7	pc (2), pv (1)
7 (R)	5	21 ± 32	32	20	pc (1), P (1), pv (1), Ca (3)
			38	26	pc (1), P (2), pv (1), Ca (3)
8 (R)	19	10 ± 8	29	3	Op (1), pv (2), I (2), ps (2), Tp (1)
			33	7	Op (2), pc (1), P (1), pv (2), I (3), ps (3), Tp (2)
			34	8	Op (3), pc (1), P (1), pv (3), I (3), ps (3), Tp (2)
9 (R)	6	14 ± 12	32	0	F (1), pc (2), P (1)
			36	4	F (1), pc (3), P (2)
10 (L)	13	14 ± 12	35	12	F (2), Fm (1), Op (2), P (1), Ca (2), pv (1), I (3), ps (1), Tp (1)
			37	14	F (3), Fm (2), Op (3), P (2), Ca (3), pv (2), I (3), ps (2), Tp (1)
11 (R)	7	9 ± 8	43	0	Fi (1), Fm (1), Ca (2), I (1), ps (1)
12 (L)	5	5 ± 7	50	0	F (1), Fi (1), Fm (1), Op (1), P (3), Ca (1), pv (3), ps (3), Tp (3)
			50	0	F (2), Fi (1), Fm (1), Op (2), P (3), Ca (1), pv (3), ps (3), Tp (3)
13 (L)	10	18 ± 13	49	24	F (2), Fi (2), Fm (2), Op (2), Ca (3), pv (1), I (2), ps (2), Tp (2), Tm (2)
			51	26	F (3), Fi (3), Fm (3), Op (3), pc (1), Ca (3), pv (2), I (3), ps (3), Tp (3), Tm (2)

Abbreviations:(1): Mild; (2): Moderate; (3): Severe; Ca: Caudate; Ci: Cingulum; F: Anterior frontal; Fi: Inferior frontal; Fm: Mesial frontal; H: Hippocampus; I: Insular; L: Left; O: Occipital; Om: Mesial occipital; Op: Opercular; P: Parietal; pc: precentral; Pm: Mesial parietal; ps: Perisylvian; pv: Periventricular; R: Right; SI: High signal intensity on T2/FLAIR; T: Temporal; T1: T1-weighted image; Ti: Trigone; Tm: Mesial temporal; Tp: Polar Temporal.

**Table 3:** Signal intensity changes in the white and in the grey matter of 13 patients with Rasmussen encephalitis. Each row represents radiological findings when any change occurred (either in atrophy or in signal intensity); first and last MR exam details are always reported. Abbreviations are the same as reported in Table 2.

Patient	MRI Timing		MRI Findings	
	Patient Age	Years from Onset	SI White Matter	SI Gray Matter
1	27	2	H (1), I (1), Tm (1)	absent
	38	13	H (1), I (1), P (3), T (1), Tm (1)	
	39	14	H (1), I (1), P (1), T (1), Tm (2)	
	42	17	H (1), I (1), P (1), T (1), Tm (1)	
	45	20	H (1), I (1), Ci (1), P (2), T (3), Tm (3)	P (2), T (1), Tm (2)
2	21	5	Fm (3), Fi (3), F (3), I (2), Tm (1) (unchanged)	F (1), I (2), Tm (1) (unchanged)
3	22	2	absent	absent
	23	3	pc (1), F (1), Fm (1)	pc (1)
	24	4	pc (2), F (2), Fm (2)	pc (2), F (1), Fm (1)
	25	5	pc (3), F (3), Fm (2)	pc (3), F (3), Fm (2)
	29	9	pc (2), F (2), Fm (2), I (2)	pc (2), F (2), Fm (2), I (2)
	37	17	pc (2), F (2), Fm (2), I (3)	pc (2), F (2), Fm (2), I (3)
4	23	8	absent (unchanged)	absent
	25	10		F (1)
5	50	2	pc (1), F (1)	pc (1)
	54	6	pc (1), F (2), I (2)	pc (1), F (1), I (1)
6	23	4	absent	pc (1)
	24	5	pc (2)	pc (2)
	26	7	absent	absent
7	32	20	P (2), Ci (2) (unchanged)	Ci (2)
	32	20		Ci (3)
	38	26		Ci (2)
8	29	3	Op (1), I (1), Tp (1)	Op (1), I (1), Tp (1)
	30	4	Op (1), I (1), Tp (1)	Op (1), I (2), Tp (1)
	31	5	Op (1), I (2), Tp (1)	Op (1), I (2), Tp (1)
	34	8	Op (2), I (2), pc (2), P (2), Tp (1)	Op (1), I (1), Tp (1), pc (1), P (1)
	44	18	Op (2), I (2), pc (3), P (2), Tp (1)	Op (1), I (1), Tp (1), pc (1), P (1)
9	32	0	P (1) (unchanged)	P (1) (unchanged)
10	35	12	F (1),Fm (2),Op (1),I (1),O (2),Om (2),ps (2)	F (1), Fm (2), Op (1), I (1), O (2), Om (2), ps (2)
	37	14	F (2), Fm (2), Op (1), I (1), O (1), Om (1), ps (2)	F (2), Fm (2), Op (1), I (1), O (1), Om (1), ps (2)
11	43	0	absent (unchanged)	Fi (2), Fm (1), I (1)
	44	1		Fi (1), Fm (1), I (1)
	47	4		absent
12	50	0	F (1),I (1),O (2),Om (2),pv (2),ps (2),Tm (2),Tp (2),Ti (2),T (2)	Om (1)
	50	0	F (2),I (2),O (2),Om (2),pv (2),ps (2), Tm (2),Tp (2),Ti (2),T (2) (stable)	absent (stable)
	52	2		
13	49	24	F (3), Fi (3), Op (3), I (2), P (1), pv (2), ps (1), Tm (1), Tp (1)	F (3), Fi (3), Fm (3), Op (3), I (3)
	51	26	F (3), Fi (3), Op (3), I (2), P (1), pv (2), ps (1), Tm (1), Tp (1)	F (3), Fi (3), Fm (3), Op (3), I (3)
	63	38	F (3), Fi (3), Op (3), I (2), P (2), pv (2), ps (1), Tm (1), Tp (1)	F (3), Fi (3), Fm (3), Op (3), I (3), P (1)

## Supplementary Materials

The following supporting information can be downloaded at: https://www.mdpi.com/article/10.3390/diagnostics12020502/s1, Table S1: detailed radiological findings.

## References

[1] Bien C.G., Tiemeier H., Sassen R., Kuczaty S., Urbach H., von Lehe M., Becker A.J., Bast T., Herkenrath P., Karenfort M. (2013). Rasmussen encephalitis: Incidence and course under randomized therapy with tacrolimus or intravenous immunoglobulins. Epilepsia.

[2] Hart Y.M., Andermann F., Fish D.R., Dubeau F., Robitaille Y., Rasmussen T., Berkovic S., Marino R., Yakoubian E.M., Spillane K. (1997). Chronic encephalitis and epilepsy in adults and adolescents: A variant of Rasmussen’s syndrome?. Neurology.

[3] Villani F., Pincherle A., Antozzi C., Chiapparini L., Granata T., Michelucci R., Rubboli G., Simone I., Bellomo R., Spreafico R. (2006). Adult-onset Rasmussen’s encephalitis: Anatomical-electrographic-clinical features of 7 Italian cases. Epilepsia.

[4] Deleo F., Matricardi S., Didato G., Pappalardo I., Villani F. (2015). The dilemma of adult-onset Rasmussen encephalitis clinical assessment: Proposal for a new bedside tool to evaluate disease progression. Epilepsy Behav..

[5] Rasmussen T., Olszewski J., Lloyd-Smith D. (1958). Focal seizures due to chronic localized encephalitis. Neurology.

[6] Varadkar S., Bien C.G., Kruse C.A., Jensen F.E., Bauer J., Pardo C.A., Vincent A., Mathern G.W., Cross J.H. (2014). Rasmussen’s encephalitis: Clinical features, pathobiology, and treatment advances. Lancet Neurol..

[7] Pardo C.A., Vining E.P.G., Guo L., Skolasky R.L., Carson B.S., Freeman J.M. (2004). The Pathology of Rasmussen Syndrome: Stages of Cortical Involvement and Neuropathological Studies in 45 Hemispherectomies. Epilepsia.

[8] Bien C.G., Bauer J., Deckwerth T.L., Wiendl H., Deckert M., Wiestler O.D., Schramm J., Elger C.E., Lassmann H. (2002). Destruction of neurons by cytotoxic T cells: A new pathogenic mechanism in rasmussen’s encephalitis. Ann. Neurol..

[9] Schwab N., Bien C.G., Waschbisch A., Becker A., Vince G.H., Dornmair K., Wiendl H. (2009). CD8+ T-cell clones dominate brain infiltrates in Rasmussen encephalitis and persist in the periphery. Brain.

[10] Schneider-Hohendorf T., Mohan H., Bien C.G., Breuer J., Becker A., Görlich D., Kuhlmann T., Widman G., Herich S., Elpers C. (2016). CD8(+) T-cell pathogenicity in Rasmussen encephalitis elucidated by large-scale T-cell receptor sequencing. Nat. Commun..

[11] Bien C.G., Granata T., Antozzi C., Cross J.H., Dulac O., Kurthen M., Lassman H., Mantegazza R., Villemure J.G., Spreafico R. (2005). Pathogenesis, diagnosis and treatment of Rasmussen encephalitis: A European consensus statement. Brain.

[12] Gitlevich T., Lado F., Moshé S. (2016). Kozhevnikov-Rasmussen Syndrome: A Historical Perspective Spanning Two Centuries. J. Pediatr. Epilepsy.

[13] Villani F., Spreafico R., Farina L., Giovagnoli A.R., Bernasconi P., Granata T., Avanzini G. (2001). Positive response to immunomodulatory therapy in an adult patient with Rasmussen’s encephalitis. Neurology.

[14] Bien C.G., Schramm J. (2009). Treatment of Rasmussen encephalitis half a century after its initial description: Promising prospects and a dilemma. Epilepsy Res..

[15] Stabile A., Deleo F., Didato G., Pastori C., Antozzi C., de Curtis M., Villani F. (2018). Adult-onset Rasmussen encephalitis treated with mitoxantrone. Eur. J. Neurol..

[16] Marras C.E., Granata T., Franzini A., Freri E., Villani F., Casazza M., De Curtis M., Ragona F., Ferroli P., D’Incerti L. (2010). Hemispherotomy and functional hemispherectomy: Indications and outcome. Epilepsy Res..

[17] Granata T., Matricardi S., Ragona F., Freri E., Casazza M., Villani F., Deleo F., Tringali G., Gobbi G., Tassi L. (2014). Hemispherotomy in Rasmussen encephalitis: Long-term outcome in an Italian series of 16 patients. Epilepsy Res..

[18] Villani F., Didato G., Deleo F., Tringali G., Garbelli R., Granata T., Marras C.E., Cordella R., Spreafico R. (2014). Long-term outcome after limited cortical resections in two cases of adult-onset Rasmussen encephalitis. Epilepsia.

[19] Chiapparini L., Granata T., Farina L., Ciceri E., Erbetta A., Ragona F., Freri E., Fusco L., Gobbi G., Capovilla G. (2003). Diagnostic imaging in 13 cases of Rasmussen’s encephalitis: Can early MRI suggest the diagnosis?. Neuroradiology.

[20] Kim S.J., Park Y.D., Pillai J.J., Lee M.R., Smith J.R. (2002). A Longitudinal MRI Study in Children with Rasmussen Syndrome. Pediatr. Neurol..

[21] Venkatesan A., Tunkel A.R., Bloch K.C., Lauring A.S., Sejvar J., Bitnun A., Stahl J.P., Mailles A., Drebot M., Rupprecht C.E. (2013). Case definitions, diagnostic algorithms, and priorities in encephalitis: Consensus statement of the international encephalitis consortium. Clin. Infect. Dis..

[22] Derry C., Dale R.C., Thom M., Miller D.H., Giovannoni G. (2002). Unihemispheric cerebral vasculitis mimicking Rasmussen’s encephalitis. Neurology.

[23] Bien C.G., Widman G., Urbach H., Sassen R., Kuczaty S., Wiestler O.D., Schramm J., Elger C.E. (2002). The natural history of Rasmussen’s encephalitis. Brain.

[24] Bhatjiwale M.G., Polkey C., Cox T.C., Dean A., Deasy N. (1998). Rasmussen’s encephalitis: Neuroimaging findings in 21 patients with a closer look at the basal ganglia. Pediatr. Neurosurg..

[25] Huang Y.-C., Weng H.-H., Tsai Y., Huang Y.-C., Hsiao M.-C., Wu C.-Y., Lin Y.-H., Hsu H.-L., Lee J.-D. (2009). Periictal magnetic resonance imaging in status epilepticus. Epilepsy Res..

[26] Meletti S., Monti G., Mirandola L., Vaudano A.E., Giovannini G. (2018). Neuroimaging of status epilepticus. Epilepsia.

[27] Larionov S., König R., Urbach H., Sassen R., Elger C.E., Bien C.G. (2005). MRI brain volumetry in Rasmussen encephalitis: The fate of affected and “unaffected” hemispheres. Neurology.

[28] Dupont S., Gales A., Sammey S., Vidailhet M., Lambrecq V. (2017). Late-onset Rasmussen Encephalitis: A literature appraisal. Autoimmun. Rev..

[29] Geller E., Faerber E.N., Legido A., Melvin J.J., Hunter J.V., Wang Z., de Chadarevian J.P. (1998). Rasmussen encephalitis: Complementary role of multitechnique neuroimaging. AJNR Am. J. Neuroradiol..

